# The CD14^+^HLA-DR^lo/neg^ Monocyte: An Immunosuppressive Phenotype That Restrains Responses to Cancer Immunotherapy

**DOI:** 10.3389/fimmu.2019.01147

**Published:** 2019-05-22

**Authors:** April E. Mengos, Dennis A. Gastineau, Michael P. Gustafson

**Affiliations:** Nyberg Human Cellular Therapy Laboratory, Department of Laboratory Medicine and Pathology, Mayo Clinic, Phoenix, AZ, United States

**Keywords:** immunosuppression, monocytes, MDSCs, cancer, immunotherapy, biomarker

## Abstract

Recent successes in cancer immunotherapy have been tempered by sub-optimal clinical responses in the majority of patients. The impaired anti-tumor immune responses observed in these patients are likely a consequence of immune system dysfunction contributed to by a variety of factors that include, but are not limited to, diminished antigen presentation/detection, leukopenia, a coordinated network of immunosuppressive cell surface proteins, cytokines and cellular mediators. Monocytes that have diminished or no HLA-DR expression, called CD14^+^HLA-DR^lo/neg^ monocytes, have emerged as important mediators of tumor-induced immunosuppression. These cells have been grouped into a larger class of suppressive cells called myeloid derived suppressor cells (MDSCs) and are commonly referred to as monocytic myeloid derived suppressor cells. CD14^+^HLA-DR^lo/neg^ monocytes were first characterized in patients with sepsis and were shown to regulate the transition from the inflammatory state to immune suppression, ultimately leading to immune paralysis. These immunosuppressive monocytes have also recently been shown to negatively affect responses to PD-1 and CTLA-4 checkpoint inhibition, CAR-T cell therapy, cancer vaccines, and hematopoietic stem cell transplantation. Ultimately, the goal is to understand the role of these cells in the context of immunosuppression not only to facilitate the development of targeted therapies to circumvent their effects, but also to potentially use them as a biomarker for understanding disparate responses to immunotherapeutic regimens. Practical aspects to be explored for development of CD14^+^HLA-DR^lo/neg^ monocyte detection in patients are the standardization of flow cytometric gating methods to assess HLA-DR expression, an appropriate quantitation method, test sample type, and processing guidances. Once detection methods are established that yield consistently reproducible results, then further progress can be made toward understanding the role of CD14^+^HLA-DR^lo/neg^ monocytes in the immunosuppressive state.

## Introduction

Exquisitely and carefully modulated immune responses coordinate the balance between preventing microbial onslaught and preventing autoimmune attack. Too little immune activation results in insufficient clearance of foreign invaders, and too much immune activation results in the targeting of self-antigens and potentially devastating autoimmune syndromes. This finely choreographed tightrope act is accomplished, in part, by a specialized array of immune cells which patrol the body and exert immunomodulatory roles. While in the larger context these cells exert the beneficial tempering of immune over-responsiveness, they can also by similar mechanisms negatively impact anti-cancer immunotherapy efficacy.

The goal of cancer immunotherapy is to successfully stimulate anti-tumor responses and overcome tumor-mediated immunosuppression. Generation of anti-tumor immunity has been accomplished through different modalities including cellular immunotherapy, vaccines, monoclonal antibodies, cytokine administration, and oncolytic virotherapy. These multi-faceted approaches have yielded tremendous clinical successes in the past few years. Even so, there have been significant difficulties in generating durable responses in a majority of cancer patients. As such, further understanding of immune dysfunction and the identification of predictive biomarkers are required so that methods may be developed to increase the efficacy of immunotherapeutic agents.

Recent data reveal that the potential exists to utilize the assessment of CD14^+^HLA-DR^lo/neg^ monocyte abundance as a biomarker to predict which patients may or may not respond to immunotherapy regimens. For CD14^+^HLA-DR^lo/neg^ monocytes in particular, an extensive array of studies involving immunotherapy demonstrate that high baseline levels of these immunosuppressive monocytes were associated with diminished anti-tumor responses and/or poor clinical outcomes. As CD14^+^ monocytes lose HLA-DR expression and thus convert from an inflammatory to an anti-inflammatory phenotype, they play a role in subverting effective anti-tumor responses, and their abundance in patient blood inversely correlates with favorable outcomes.

The purpose of this review is to highlight the documented findings which demonstrate this correlation. In the context of cancer immunotherapy, the abundance of these cells may guide patient selection and/or provide patient monitoring capabilities for understanding clinical responses on the individual level. Since there are considerable differences in the biology of these cells in animal models compared to human studies, this review will focus mainly on published data from human studies and clinical trials. However, in some cases, examples may be provided from animal models where the observations appear to be congruent with human data.

## CD14^+^HLA-DR^lo/neg^ Monocytes are Immunosuppressive Cells That Respond to Systemic Pro-Inflammatory Conditions

Myeloid cells that suppress the immune system have been described by a variety of names including myeloid derived suppressor cells (MDSCs), M2 monocytes/macrophages, tumor associated macrophages/myeloid cells, and regulatory myeloid cells (1–3). They are a heterogeneous population comprised of precursors of granulocytes, macrophages, and dendritic cells (DC). However, their characterization and classification into different subsets remains to be resolved as there are considerable inconsistencies in the way these subsets are defined and reported (4). Monocytes that have low or no HLA-DR expression have been most commonly referred to as CD14^+^HLA-DR^lo/neg^ monocytes or monocytic MDSCs. HLA-DR is one of three MHC class II glycoproteins expressed on antigen-presenting cells whose function is to present peptides derived from antigens ingested by the cell to T-cell receptors (TCR) resulting in T-cell activation. As such, these cells have a diminished capacity to present antigens to T cells and a large body of work has demonstrated these cells to be immunosuppressive. Since the functional capacities related to the immunosuppressive mechanisms of these cells have been reviewed elsewhere (5–7), this subject will not be discussed here. Although, there is still considerable debate over the origins of human MDSCs (8), several lines of evidence that will be discussed in this review suggest that CD14^+^HLA-DR^lo/neg^ monocytes should be best understood in terms of arising from the normal circulating monocyte pool and not from an early precursor cell independent of monocytes. As for other MDSCs, lineage negative (CD3^−^CD19^−^CD56^−^CD14^−^) LIN^−^CD33^+^HLA-DR^−^ cells have been described as immature MDSCs (iMDSCs) and CD33^+^CD15^+^HLA-DR^−^ cells as polymorphonuclear MDSCs (PMN-MDSCs). CD14^+^HLA-DR^lo/neg^ monocytes also express high levels of CD33 and CD11b on their surface. As will be discussed later, CD33 expression is greater on monocytes compared to other myeloid cells. CD11b is expressed on nearly all myeloid cells but also is expressed on human natural killer NK cells (9) and therefore is not an appropriate marker for human MDSCs. For the sake of brevity, CD33^+^CD11b^+^CD14^+^HLA-DR^lo/neg^ immunosuppressive monocytes throughout this review will be referred to as CD14^+^HLA-DR^lo/neg^ monocytes/cells. It should also be noted that there has been another type of immunosuppressive monocyte described as CD1c/BDCA1^+^CD14^+^ (10). These CD1c^+^ monocytes are a mix of classical and intermediate monocytes and are functionally distinct from CD1c^+^ dendritic cells (11). These cells express HLA-DR but not to the same extent as dendritic cells (10, 11).

Monocytes play a critical role in the response to infection. Sepsis results when the initial strong pro-inflammatory phase [referred to as systemic inflammatory response syndrome (12, 13)] then switches to an anti-inflammatory phase. At first glance, it appears that these conditions act sequentially, but there are likely elements of both pro- and anti- inflammatory mediators throughout the entire process. Monocytes are highly sensitive in the transition to the immunosuppressive state and become deactivated, resulting in a phase known as “immunoparalysis” (14, 15) or compensatory anti-inflammatory response syndrome (16, 17). Immunoparalysis is defined by a decrease in the level of HLA-DR expression on monocytes during the course of sepsis.

One group has defined immunoparalysis in patients with septic shock as having occurred when < 30% of the monocyte pool expresses HLA-DR (14). In the early stage of sepsis in these patients, pro-inflammatory cytokines such as TNFα, IL-1, GM-CSF, and IL-6, drove the deactivation of monocytes by down regulating HLA-DR through IL-10 and TGF-β mediated pathways and diminished capacity for pro-inflammatory cytokine production (16, 18). These observations have been confirmed by many other studies. For example, in patients with injuries from blunt trauma, those that had low levels of HLA-DR on monocytes after the second day of admission were significantly more likely to develop sepsis than those patients that had high levels (19). Monneret et al. demonstrated the relationship of low HLA-DR levels to survival in patients with sepsis (20). In the early stages of septic shock, the expression of HLA-DR on monocytes was not different between survivors and non-survivors. However, after 48 h post onset, survivors had significantly higher expression of HLA-DR on monocytes than those that did not survive the event.

In contrast, there are some reports that have not found a relationship between the loss of monocyte HLA-DR expression and septic shock (21, 22). These apparent contradictory reports perhaps may result from differences in the timing of sample procurement, clinical settings, and measurement parameters for HLA-DR expression or other uncharacterized variables. However, a recent review has confirmed the regulatory role of CD14^+^HLA-DR^lo/neg^ monocytes in both normal and pathological responses to a diverse array of microbial infections (23).

The loss of HLA-DR on monocytes has been reported in other non-malignant conditions with an inflammatory component. CD14^+^HLA-DR^lo/neg^ monocytes have been described in patients with severe burns (24, 25), acute and chronic liver inflammation (26–28), pancreatitis (29–31), amyotrophic lateral sclerosis (32), and immediately after surgical procedures (33). Although the precise mechanisms of monocyte deactivation and HLA-DR loss have yet to be elucidated in each of these diseases, a familiar pattern of either acute or chronic inflammation tends to be an initial event triggering the development of immunosuppressive monocytes. Taken together, the overall data in non-malignant conditions demonstrate that the loss of HLA-DR is a well-established marker of functional deactivation of monocytes and that it associates with poor clinical outcomes in critically ill patients.

HLA-DR can be down regulated through a variety of mechanisms. Under normal physiological conditions, HLA-DR is under the transcriptional control of the MHC Class II transactivator (CIITA) (34, 35). HLA-DR expression can be induced by IFN-γ through transcriptional activation via CIITA (36) and also by GM-CSF possibly through post-transcriptional mechanisms (33). Conversely, several cytokines can down-regulate monocytic HLA-DR expression. IL-1β and TGF-β directly down-regulate transcription of HLA-DR through CIITA and/or prevent IFN-γ induction of HLA-DR (16, 37, 38). IL-10 also strongly decreases surface HLA-DR expression but it's mechanism of action is to increase intracellular sequestration of MHC Class II molecules (39) via ubiquitination by inducing the membrane-associated RING-CH (MARCH) ubiquitin ligase (40). Glucocorticoids and steroid hormones can also negatively regulate HLA-DR transcription by decreasing CIITA mRNA levels. Down-regulation of HLA-DR on monocytes has been observed in response to cortisol (41), prednisolone (42), and dexamethasone (43, 44). Overall, many of these mechanisms that regulate HLA-DR expression have been shown to be critical mediators of immune paralysis in both sepsis and in malignant settings.

Soon after the role of monocyte deactivation was observed in sepsis patients, reports began appearing in the literature that cancer patients also exhibit monocytes with low HLA-DR expression. Patients with glioblastoma (45), ovarian cancer (46), and melanoma (47) were some of the first cancer patients discovered to have low monocytic HLA-DR expression. In previous studies with our colleagues at Mayo Clinic Rochester, we found high levels of these cells in a variety of different cancer patient groups including those with glioblastoma (43), non-Hodgkin lymphoma (48), chronic lymphocytic leukemia (49), and renal cell carcinoma (50). In order to understand the severity of immunosuppression in cancer patients, we compared the presence of CD14^+^HLA-DR^lo/neg^ monocytes in cancer patients to those patients with acute lung injury with or at risk for sepsis (51). Many of the cancer patients had levels of CD14^+^HLA-DR^lo/neg^ monocytes equally high as patients with sepsis.

*In vitro* experiments demonstrated that monocytes isolated from healthy volunteers can lose HLA-DR expression through co-culture with tumor-derived exosomes (47), exposure to conditioned media from cultured tumor cells (52, 53), or even incubation with cytokines like TGF-β (37). Furthermore, Ribechini et al. have identified a potentially unique pathway in which GM-CSF can license CD14^+^ monocytes such that upon later exposure to INF-γ, the monocytes would switch to an immunosuppressive phenotype through the upregulation of indolamine 2,3-dioxygenase (IDO) (54). Bergenfeltz et al. found that monocytes isolated from breast cancer patients exhibited gene expression profiles similar to monocytes isolated from sepsis patients (55). Specifically, TNFα, IL-1β, HLA-DR, and CD86 genes were significantly down-regulated in monocytes from breast cancer patients compared to controls suggesting that some of the mechanisms that convert monocytes to the immunosuppressive state are identical in both septic and malignant conditions.

The implications of these findings for cancer immunotherapy are significant. The presence of high levels of CD14^+^HLA-DR^lo/neg^ monocytes suggests that many of these cancer patients had reached a point of immunoparalysis prior to treatment and thus may not be very responsive to immunotherapeutic approaches. On the other hand, many cancer patients have been observed with normal levels of CD14^+^HLA-DR^lo/neg^ monocytes. The timing of onset, progression and intensity of immunoparalysis in cancer patients compared to patients with sepsis will certainly involve both similar and unique mechanisms. As such, further work is needed to understand how these cells respond and contribute to tumor development.

## Impact on Immunotherapy

### Checkpoint Inhibitors

The impact of CD14^+^HLA-DR^lo/neg^ monocytes on CTLA-4 inhibition with ipilimumab has most clearly been demonstrated in melanoma patients with advanced disease. Meyer et al. reported that CD14^+^HLA-DR^lo/neg^ monocytes were elevated in melanoma patients. While CD14^+^HLA-DR^lo/neg^ monocyte populations were not affected by ipilimumab treatment, patients that responded to ipilimumab treatment had significantly less pre-treatment frequencies of CD14^+^HLA-DR^lo/neg^ monocytes than those patients that did not respond to treatment (56). In another study, lower pre-treatment frequencies of CD14^+^HLA-DR^lo/neg^ monocytes were associated with overall patient survival (57). The percentages of CD14^+^HLA-DR^lo/neg^ cells of total monocytes appeared to be more predictive of survival than absolute cell counts (cells/μl). The authors also reported that after 6 weeks of ipilimumab treatment, lower percentages of CD14^+^HLA-DR^lo/neg^ cells were associated with higher changes in absolute T cell counts, suggesting that the CD14^+^HLA-DR^lo/neg^ monocytes restricted CD8^+^ T cell response. These data were confirmed to some extent by Tarhini et al. (58), Martens et al. (59) and Gebhardt et al. (60). Gebhardt et al. found that decreased CD14^+^HLA-DR^lo/neg^ monocytes were related to declines in nitric oxide production in response to ipilimumab treatment. Finally, de Coaña et al. found that in melanoma patients PMN-MDSCs decreased upon ipilimumab treatment whereas CD14^+^HLA-DR^lo/neg^ monocytes did not change (61). However, in patients who received a clinical benefit, CD14^+^HLA-DR^lo/neg^ monocytes decreased after treatment whereas this was not the case in patients who progressed. While the frequency of CD14^+^HLA-DR^lo/neg^ monocytes was not compared to healthy volunteers, baseline levels of these cells were similar between patients with progressive disease and those that had a clinical benefit. Taken together, the results from these studies present an interesting dynamic. Clearly, lower baseline frequencies of CD14^+^HLA-DR^lo/neg^ monocytes are predictive of outcome and therefore these monocytes may interfere with the efficacy of ipilimumab treatment. However, in some patients, particularly for those who do respond to treatment, there is evidence that immunosuppressive monocytes decline after treatment. Further studies are needed to confirm and delineate the mechanisms behind these observations.

Data are also emerging that demonstrate the role of monocytes in altered responses to anti-PD-1 therapy. In a study of stage IV melanoma patients receiving anti-PD-1 therapy, single cell mass cytometry was used to investigate peripheral blood biomarkers (62). The pre-treatment frequency of classical monocytes (CD14^+^CD16^−^) that express high levels of HLA-DR was predictive of overall survival in these patients. The majority of CD14^+^HLA-DR^lo/neg^ monocytes are classical monocytes so the higher expression of HLA-DR in these patients likely reflects lower levels of immunosuppressive monocytes. The authors of this study did report that a population similar to CD33^lo^CD11b^+^HLA-DR^lo^ cells was not different between responders and non-responders. However, the gating strategy for isolating CD33^lo^CD11b^+^HLA-DR^lo^ cells could very well include both monocytic and granulocytic populations. In mixed lymphocyte reaction proliferation assays using PBMCs from healthy donors, it was shown that proliferation of nivolumab-treated T cells improved in the absence of monocytes (63). Additionally, blockade of CSF-1R signaling on monocytes improved T cell proliferation. Interestingly, the authors of this study also report that activation of T cells by nivolumab treatment induced the release of M-CSF from T cells thereby increasing the immunosuppressive functions of monocytes through adenosine production and upregulation of PD-L1 on monocytes. PD-1 and CSF-1R blockade in combination was also found to augment the cytolytic capacity of tumor infiltrating lymphocytes (TILs) in co-cultures of CD3^+^ TILs and CD11b^+^ tumor infiltrating myeloid cells from patients with glioblastoma (64). CD14^+^HLA-DR^lo/neg^ monocyte levels also appear to distinguish responders from non-responders in nivolumab treated metastatic melanoma patients who progressed after ipilimumab therapy (65). Since there have not been many mechanistic insights garnered from these human studies, further investigation is needed to determine whether the impact of CD14^+^HLA-DR^lo/neg^ monocytes on checkpoint inhibition reflects a general immunosuppressive environment or to what degree the expression of monocytic PD-1 and/or PD-L1 disrupts the efficacy of checkpoint blockade (66–68).

### Cancer Vaccines

Data from studies and clinical trials demonstrate that immunosuppressive monocytes impact cancer vaccines through several mechanisms that limit optimal anti-tumor responses. They have been shown to adversely affect responses to direct injection of peptides/whole tumor cells as well as to *ex-vivo* differentiated dendritic cell (DCs) vaccines. In a therapeutic cancer vaccine study using multiple tumor-associated peptides (TUMAPs) in renal cell carcinoma (RCC) patients, Walter et al. looked at six populations of MDSCs (among other immune parameters) to determine whether these cells influenced the survival outcome of patients treated with the vaccine (69). Although five of the six types of MDSCs were elevated in RCC patients prior to treatment, CD14^+^HLA-DR^lo/neg^ monocytes demonstrated the strongest association with overall survival whereby their presence in high numbers was inversely correlated with survival. In non-small cell lung cancer (NSCLC) patients receiving a telomerase peptide vaccine, high levels of CD14^+^HLA-DR^lo/neg^ monocytes were found to be associated with poorer progression free survival (70). Prostate cancer patients with low frequencies of CD14^+^HLA-DR^lo/neg^ monocytes prior to receiving prostate/GVAX vaccine in combination with ipilimumab had a mean survival time of 52 months compared to 20 months mean survival for those with high pre-treatment frequencies (71). These cells did increase during the course of treatment but the increase did not correlate with response to treatment. In a trial testing DCs for patients with primary recurrent glioblastoma, both CD15^+^SSC^lo^ cells and CD14^+^HLA-DR^lo/neg^ monocytes were found to be elevated in patients that progressed but only the CD14^+^HLA-DR^lo/neg^ monocytes were found to be both higher as a percent of parent populations and also in cell counts (cells/μl) (72). Poschke et al. demonstrated in stage IV melanoma patients that the presence of CD14^+^HLA-DR^lo/neg^ cells purified with elutriated monocytes negatively impacted the maturation, migration, antigen uptake, and cytokine production of DCs (73). In another trial with melanoma patients, BDCA1^+^CD14^+^ cells were shown to inhibit T cell proliferation in an antigen-dependent fashion resulting in impaired responses to monocyte-derived DCs (10). In pre-clinical studies, our group has found that monocytes purified by immunomagnetic selection from a variety of cancer patients have deficiencies in DC maturation and that the method of cell culture can influence their maturation (7). Additionally, we found that CD14^+^HLA-DR^lo/neg^ cells inversely correlated with the maturation marker CD83 on dendritic cells. Taken together, the data from these studies and clinical trials demonstrate that immunosuppressive monocytes impact cancer vaccines through several mechanisms that limit optimal anti-tumor responses.

### Hematopoietic Progenitor Cell Transplantation

The functional consequences of immunosuppressive monocytes in hematopoietic progenitor cell transplantation appear to be dependent on the type of transplant. In autologous transplants, higher levels of CD14^+^HLA-DR^lo/neg^ cells in leukapheresis products were independent predictors of adverse outcomes both in terms of overall survival and progression free survival in patients with non- Hodgkin lymphoma (74). However, in the allogeneic setting, CD14^+^HLA-DR^lo/neg^ cells may confer protection against acute graft vs. host disease (aGVHD). Myeloid cells, including CD14^+^HLA-DR^lo/neg^ cells, are some of the first cells to recover after transplantation (75, 76). Mougiakakos et al. demonstrated that transplant patients had both elevated monocytes and CD14^+^HLA-DR^lo/neg^ cells at 1–3 months post-transplant (77). They also demonstrated that higher frequencies in peripheral blood also associated with higher grades of aGVHD. The induction of these cells was likely in response to circulating levels of G-CSF and IL-6, along with other pro-inflammatory cytokines. The administration of G-CSF alone to human donors was sufficient to expand both CD14^+^HLA-DR^lo/neg^ monocytes and PMN-MDSCs in both phenotype and function (78). Similarly, the presence of high CD14^+^HLA-DR^lo/neg^ monocyte cell counts in the G-CSF mobilized graft was associated with lower risks of developing aGVHD in recipients without affecting the relapse rate or the transplant-related mortality rate (79). So whereas immunosuppressive monocytes negatively affect outcomes in autologous transplant patients, they conversely may provide a benefit of a reduced risk of aGVHD in allogeneic transplant recipients.

### Adoptive and Chimeric Antigen Receptor (CAR) T Cell Therapies

CD14^+^HLA-DR^lo/neg^ monocytes may negatively impact the effectiveness of adoptive and Chimeric Antigen Receptor (CAR) T cell therapies. While the data from clinical trials is too limited to show this conclusively, the ability of suppressive monocytes to inhibit T cells in human cancers has been well documented (43, 48, 80–83) and has been associated both with dysfunctional antigen-specific T cells and negative outcomes in melanoma patients (84). CD19-CAR T cell expansions from mononuclear cell collections that contained high percentages of monocytes produced poorer cell yields in children with acute lymphocytic leukemia and non-Hodgkin lymphoma (85). While they did not specifically measure CD14^+^HLA-DR^lo/neg^ monocytes, when they depleted monocytes by adherence to plastic techniques, cell expansion improved, and typical expected yields were achieved. While data has yet to be published from clinical trials monitoring immunosuppressive monocytes in those receiving CAR-T cell therapies, reports from animal model studies suggest that rodent MDSCs are expanded after treatment with CAR-T cells in a GM-CSF dependent fashion, and that this limited the anti-tumor activity of the infused cells (86). Other data from animal models suggest that although transferred T cells likely induce myeloid derived suppressor cells (87), there may be some conditions in which T cell therapy can be successful despite immunosuppression caused by myeloid cells (88). As data emerges in the human setting, it is not unreasonable to expect that CD14^+^HLA-DR^lo/neg^ monocytes will be shown to play some role in reducing the anti-tumor efficacy of CAR T cells because the nature of the cytokine release syndrome involves pro-inflammatory cytokines which have previously been demonstrated to induce these cells under other conditions.

## Efficacy of Therapeutic Approaches Targeting Immunosuppressive Monocytes

As evidence for the role of immunosuppressive monocytes in inhibiting anti-tumor responses continues to build, it becomes readily apparent that therapeutically targeting these cells should improve responses to immunotherapy. Agents designed to interfere with MDSCs have generally been classified into four categories: (1) inhibition of the conversion, appearance and/or expansion of MDSCs, (2) inhibition of MDSC immunosuppressive functions, (3) interference of MDSC trafficking to tumors, and (4) direct removal and cytotoxic approaches (89–91). Several agents that interfere with these mechanisms have shown promise in pre-clinical animal studies and have been reviewed elsewhere (89–93). As these drugs move into clinical trials, it will be very important understand how they will affect each of the different MDSC populations as well as total myeloid cells.

Several examples in the literature which are summarized in Table 1 highlight how drugs targeted to MDSCs affect the subpopulations in a differential manner. For example, gemcitabine has been shown to reduce MDSC accumulation in tumors in animal models (94) but appears to preferentially decrease PMN-MDSCs and total monocytes but not monocytic MDSCs when tested in pancreatic cancer patients (95). In another case, the treatment of solid tumor cancer patients with an agonistic TRAIL-R2 antibody resulted in the decline of different MDSC populations while not affecting other myeloid populations (96). Tyrosine kinase inhibitors (TKI) also demonstrate differential effects on MDSCs. In chronic myeloid leukemia patients treated with imatinib, nilotinib, or dasatinib, all three TKIs decreased PMN-MDSCs but only dasatinib reduced CD14^+^HLA-DR^lo/neg^ monocytes (97). Furthermore, the decline in CD14^+^HLA-DR^lo/neg^ monocytes correlated with positive patient molecular responses. Another TKI, sunitinib, was shown to preferentially inhibit the suppressive activity of CD14^+^CD16^+^ monocytes and reductions in these cells were associated with sunitinib responders (98).

**Table 1:** Therapeutic approaches targeting immunosuppressive monocytes.

**Agent**	**Mode of action**	**Observed effects on human MDSCs**
Gemcitabine	Inhibition of the expansion of MDSCs	• Preferentially decreases PMN-MDSCs and total monocytes but not monocytic MDSCs in pancreatic cancer patients
TRAIL-R2 antibody	Apoptotic programmed cell death	• Declines in different MDSC populations while not affecting other myeloid populations
Tyrosine kinase inhibitor (TKI) imatinib	Interference with signal transduction, suppressing cell proliferation, differentiation, migration, metabolism and programmed cell death	• Decreases PMN-MDSCs
Tyrosine kinase inhibitor (TKI) nilotinib	Interference with signal transduction, suppressing cell proliferation, differentiation, migration, metabolism and programmed cell death	• Decreases PMN-MDSCs
Tyrosine kinase inhibitor (TKI) dasatinib	Interference with signal transduction, suppressing cell proliferation, differentiation, migration, metabolism and programmed cell death	• Decreases PMN-MDSCs • Decreases CD14+HLA-DR^lo/neg^ monocytes, which correlates with positive patient molecular responses.
Tyrosine kinase inhibitor (TKI) sunitinib	Interference with signal transduction, suppressing cell proliferation, differentiation, migration, metabolism and programmed cell death	• Preferentially inhibits suppressive activity of CD14+CD16+ monocytes, which associates with sunitinib responders
Small molecule CCR2 inhibitor PF-04136309	Prevention of monocyte trafficking to tumors (likely including immunosuppressive monocytes)	• Improves anti-tumor immunity
CSF-1R inhibitor PLX3397	Myeloid differentiation, monocytic commitment, trafficking, survival, and proliferation of monocytes and macrophages	• In recurrent glioblastoma, percentage of non-classical monocytes (CD14^lo^CD16^+^) declines but microglia in the tumor is only modestly reduced. (However other glioblastoma subtypes may respond differently)
CSF-1R inhibitor BLZ945	Myeloid differentiation, monocytic commitment, trafficking, survival, and proliferation of monocytes and macrophages	• In co-culture with neuroblastoma, monocytes partially recover HLA-DR and CD86 expression, reducing immunosuppression of T-Cell proliferation
GM-CSF	Stimulation of myelopoiesis and promotion of anti-tumor immunity	• Reverses monocyte deactivation in sepsis by inducing HLA-DR expression • Utility in cancer patients remains to be determined. • May also promote the development of CD14^+^HLA-DR^lo/neg^ monocytes, dependent on dosing, timing, and other mechanisms.

*References provided in the text*.

There have been a few clinical trials that have specifically targeted monocytes and/or immunosuppressive monocytes. Nywening et al. hypothesized that pharmacological prevention of monocyte trafficking to tumors via a small molecule CCR2 inhibitor improves anti-tumor immunity (99). They tested the CCR2 inhibitor PF-04136309 in combination with FOLFIRINOX chemotherapy vs. chemotherapy alone in patients with pancreatic ductal adenocarcinoma. The CCR2 inhibitor prevented monocyte egress from bone marrow and subsequently reduced monocyte infiltration into tumors. The reduced monocyte infiltrate resulted in an increase of intra-tumoral lymphocytes and improved anti-tumor immunity. Patients receiving the combination of PF-04136309 and chemotherapy had higher than expected response rates. One potential caveat is that monocyte blockade may result in increased infiltration of granulocytes and, consequently, dual blockade of both monocytes and granulocytes has been proposed (100). While this study did not measure immunosuppressive monocytes *per se*, there is ample evidence that immunosuppressive monocytes also migrate to tumors via the CCL2/CCR2 pathway (43, 50, 101, 102).

In other clinical trials, investigators have tested the effectiveness of blocking CSF-1R signaling in cancer patients. Myeloid differentiation, monocytic commitment, trafficking, survival, and proliferation of monocytes/macrophages are all influenced by CSF-1R signaling (103). It is hypothesized that blocking the signaling function of this receptor will result in the reduction of monocyte/macrophage infiltration into tumors and consequently limit the immunosuppressive nature of the tumor microenvironment (104). In patients with recurrent glioblastoma treated with an oral CSF-1R inhibitor PLX3397, the percentage of non-classical monocytes (CD14^lo^CD16^+^) declined after treatment but microglia in the tumor microenvironment were only modestly reduced (105). However, while taking this into account, it may be that different glioblastoma subtypes (i.e., pro-neural glioblastoma) may be more susceptible to the reprogramming of monocytes/macrophages from CSF-1R inhibition (106). Finally, CSF-1R^+^ myeloid cells are associated with negative outcomes in neuroblastoma patients (107). In monocytes co-cultured in the presence of neuroblastoma cells, the CSF-1R inhibitor BLZ945 partially restored HLA-DR and CD86 expression and reduced the immunosuppressive capacities of the monocytes on T cell proliferation.

GM-CSF has been used to support myelopoiesis and promote anti-tumor immunity as a stand-alone monotherapy and also to complement various immunotherapeutic approaches (108). Although GM-CSF has been shown to overcome monocyte deactivation in sepsis by inducing HLA-DR expression, the utility for use in cancer patients remains to be determined. In some cases, GM-CSF has been shown to provide a clinical benefit (109–112). But in several instances, GM-CSF has demonstrated neutral or negative results (113, 114). While it is clear that GM-CSF can act via pro- and anti- inflammatory pathways, emerging data, more prevalent in mouse models (115–119) but also from human studies (80, 120), indicate that GM-CSF strongly promotes the development of CD14^+^HLA-DR^lo/neg^ monocytes. The data from Ribechini et al. suggest that the timing of GM-CSF administration may be critical in the transitioning of pro-inflammatory to anti-inflammatory pathways (54). Therefore, in order to optimize GM-CSF therapy, it is critical to further understand and define how dosing, timing, and other mechanisms contribute to CD14^+^HLA-DR^lo/neg^ monocyte accumulation.

A common theme emerges from all studies cited. Therapies targeting immunosuppressive monocytes have a wide variety of effects on these cells, and underlying mechanisms are still not well understood. While many of these studies remain relatively limited in scope, and much work remains to better identify the optimal strategies and indications, these promising preliminary results clearly warrant further investigation into developing methods to target monocytes in cancer patients. The negative effect of immunosuppressive monocyte levels, particularly of the CD14^+^HLA-DR^lo/neg^ phenotype, is clear. Therefore, rigorous and well-defined immune monitoring and phenotyping of patient myeloid cells in clinical trials is justified, as their measurement is critical for understanding the mechanism(s) of action of such therapies.

## The Potential for Utilizing Immunosuppressive Monocytes as a Predictive Biomarker

The clinical significance of the broad class of MDSCs has been well documented and the pathway to utilizing these cells as biomarkers has recently been proposed (121). In many studies, the presence of CD14^+^HLA-DR^lo/neg^ monocytes in circulation has been shown to be a systemic marker of immune suppression, and has been associated with the accumulation of these cells in tumors (50). Both their ability to impair anti-tumor immune responses and that they may be a promising therapeutic target make a compelling case for the development of standardized tools and/or assays to measure CD14^+^HLA-DR^lo/neg^ monocytes in a manner that is useful for guiding therapeutic decisions for patients receiving immunotherapy.

Perhaps the simplest and most efficient way to measure CD14^+^HLA-DR^lo/neg^ monocytes is by flow cytometry of peripheral blood; therefore, acquisition of tumor biopsies which may not always be available from patients is unnecessary. The small sample of blood that is required to measure these cells justifies further investigation into monitoring them as an informative biomarker. Significant variability currently exists in the way these cells have been measured and reported in the literature. We outline these variables in Table 2 and highlight areas of concern, including differences in flow cytometry gating strategies, cell enumeration methods, timing of sample procurement, and processing procedures. These differences in methodology have been problematic, creating variability in actual, and reported results. Mandruzzato et al. have shown that the lack of standardized gating strategies was one of the largest factors of variation when measuring the total group of MDSCs (122). Nonetheless, standardized gating strategies have been used to gain meaningful correlations to clinical outcomes. We and others (124) have shown that standardization of the measurement of these cells can result in consistent and robust assays.

**Table 2:** Recommendations for consistent and reproducible reporting for immunosuppressive monocytes by flow cytometry.

**Processing steps that contribute to variation in reporting**	Examples observed in the literature^*^	**Recommendations for best practices**
Phenotypes: combinations reported (2, 122, 123)	CD14^+^HLA-DR^lo/neg^ CD33^+^HLA-DR^−^ LIN^−^, CD14^+^, HLA-DR^−^ CD33^+^, CD11b^+^, CD14^+^	CD33 (bright): confirm myeloid origin CD14 (+): parent population HLA-DR (lo/neg): distinguish between “normal” and immunosuppressive populations CD16: identify subpopulations
Processing of blood samples	Purification of mononuclear cells/Ficoll separation Effects of cryopreservation/thawing Timing of sample collection and storage	Directly stain whole blood Blood draws taken at approximately same time of day (i.e., mornings) Samples processed within 4–6 h; held at room temperature
Quantification/enumeration of CD14^+^HLA-DR^lo/neg^ cells	As % of PBMCs As % of Total Leukocytes Cells/μl HLA-DR MFI	Cells/μl % of CD14 Molecules per cell
Gating strategies	Monocyte measurement by Forward and Side scatter CD14^+^ from Mononuclear gate Histogram or Quadrant of HLA-DR expression	CD33^br^ from Mononuclear gate, then CD14^+^ cells Fluorescence minus one (FMO) staining to determine HLA-DR negative cells

* *Not all inclusive*.

**Figure 1:**
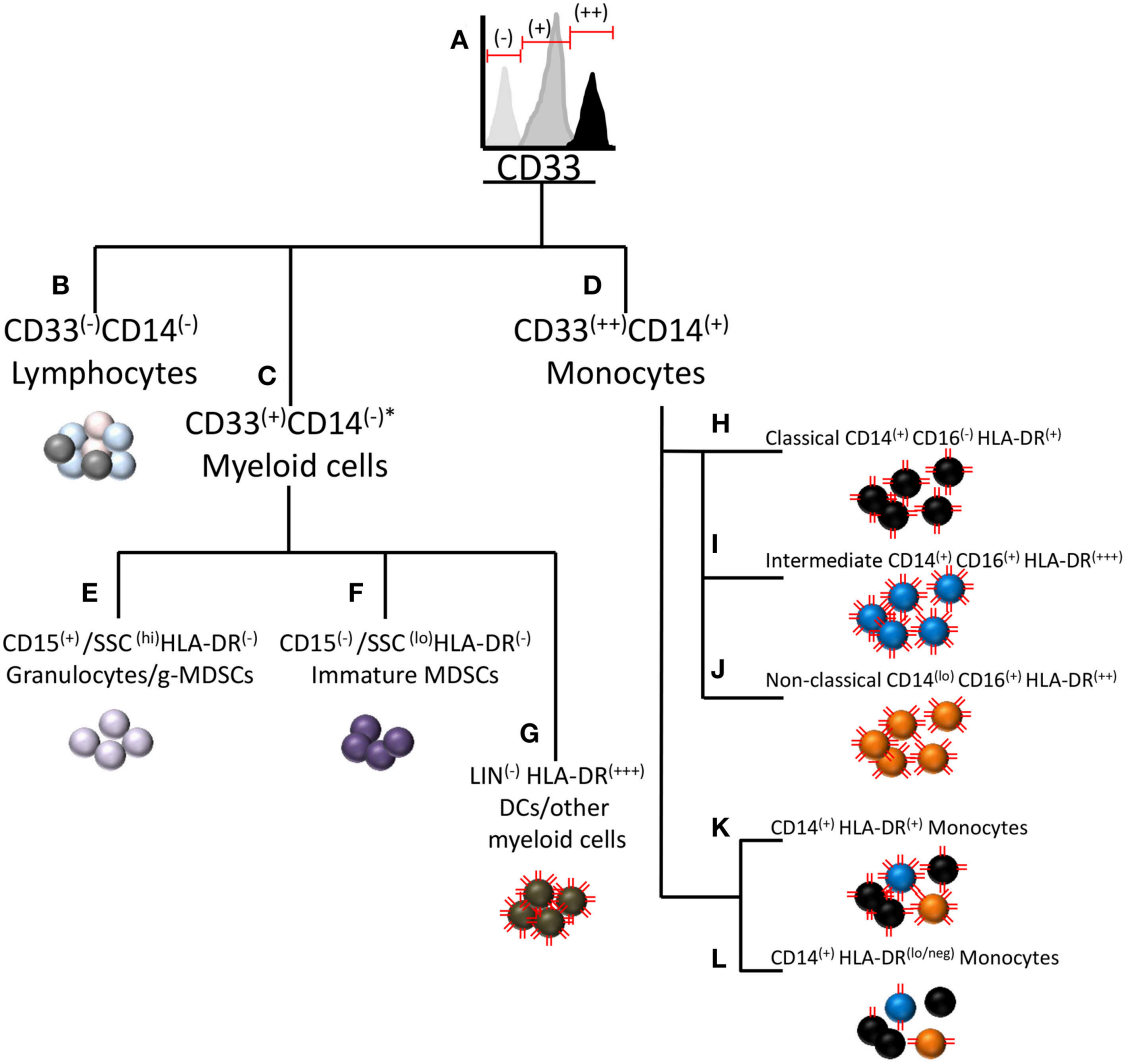
Relationship of immunosuppressive CD14^+^HLA-DR^lo/neg^ monocytes to other myeloid cells and myeloid derived suppressor cells. A diagram of CD marker commonalities between cell types within the myeloid subclass including MDSCs and other cells with similar CD marker expression. **(A)** MDSCs of both granulocytic and monocytic lineage reside within the CD33^+^ population of cells. **(B)** Cells not expressing CD33 are considered to be of lymphoid lineage. **(C)** Cells not expressing CD14 comprise a separate pool of CD33^+^ subtypes. **(D)** CD14^+^ monocytes typically strongly express CD33, hereafter noted as CD33^(++)^. **(E)** CD15^(+)^/SSC^(hi)^/HLA-DR^(−)^ normal granulocytes and granulocytic MDSCs. **(F)** CD15^(−)^/SSC^(lo)^/HLA-DR^(−)^ immature MDSCs. **(G)** LIN^(−)^ HLA-DR^(+++)^ dendritic cells or other myeloid cells. Three sub-populations of monocytes are **(H)** classical monocytes (CD14^+^CD16-) **(I)** intermediate monocytes (CD14^+^CD16^+^) **(J)** Non-classical monocytes (CD14^lo^CD16^+^) **(K)** Representation of the combined pool of the three monocyte subtypes **(L)** The CD14^+^HLA-DR^lo/neg^ cell most typically arises from the classical monocyte pool, but may also be derived from the intermediate and non-classical monocyte pools. The red lines on the cells represent relative HLA-DR expression on the cells. ^*^Under some conditions, granulocytes can express CD14 (126).

Typically, CD14^+^HLA-DR^lo/neg^ monocytes are measured by flow cytometry in blood samples collected from patients. These monocytes are phenotypically positive for CD14, CD33, and CD11b (125). The source of inconsistencies is often in the measurement of monocyte HLA-DR loss, as HLA-DR expression exhibits considerable variation and is not uniform across all subtypes of monocytes. Figure 1 displays a diagram of surface marker commonalities within the myeloid compartment. CD33 expression on human myeloid cells appears to be bi-modal as granulocytes and immature myeloid cells express moderate amounts of CD33 whereas monocytes exhibit strong CD33 expression (Figure 1A). MDSCs of both granulocytic and monocytic lineage reside within the CD33^+^ population of cells. CD33^−^ cells comprise cells from the lymphoid lineage (Figure 1B). Often CD33 and HLA-DR are used to measure MDSCs but these two markers are not solely sufficient to distinguish the three types of MDSCs. To distinguish monocytic MDSCs by flow cytometry, CD33 positive cells are gated from total leukocytes and then monocytes are further gated based on CD14 expression. The combination of CD33^++^ and CD14^+^ distinguishes monocytes from all other myeloid cells (Figure 1D). CD33^+^ cells not expressing CD14 comprise a separate pool of CD33^+^ subtypes (Figure 1C) including CD15^(+)^/SSC^(hi)^/HLA-DR^(−)^ granulocytes and granulocytic MDSCs (currently very few reproducible markers distinguish g-MDSCs by flow cytometry) (Figure 1E), CD15^(−)^/SSC^(lo)^/HLA-DR^(−)^ immature MDSCs (Figure 1F), and LIN^(−)^ HLA-DR^(+++)^ dendritic cells or other myeloid cells (Figure 1G). Although the illustration is meant to visualize the hierarchy of myeloid cell populations, it is likely that some myeloid progenitors may become CD14^+^ and hence join the pool of monocytic cells.

The immunosuppressive monocyte of phenotype CD14^+^HLA-DR^lo/neg^ resides within the CD33^(++)^/CD14^(+)^ population of mononuclear cells (Figure 1D). This population may be further sub-divided based on CD16 expression into classical (CD14^+^CD16^−^), intermediate (CD14^+^CD16^+^), and non-classical (CD14^lo^CD16^+^) monocytes [Figures 1H–J and Ziegler-Heitbrock et al.(127)]. Interestingly, HLA-DR expression varies between these subgroups in that intermediate monocytes express the highest amount of HLA-DR and classical monocytes expressing the least (125). Note that the HLA-DR expression level noted as HLA-DR^(+)^, is not low enough to classify as the immunosuppressive HLA-DR^(lo/neg)^ phenotype. From the combined pool of the three monocyte subtypes (Figure 1K), the immunosuppressive phenotype may arise by loss of HLA-DR expression (Figure 1L). The CD14^+^HLA-DR^lo/neg^ cell most typically arises from the classical monocyte pool, but may also be derived from the intermediate and non-classical monocyte pools. For setting the HLA-DR^(+)^ vs. HLA-DR^(lo/neg)^ threshold in flow cytometry, a convenient internal negative control is available in the CD33^−^CD14^−^HLA-DR^−^ mononuclear cell population. The threshold is set at the upper limit of HLA-DR in that population, thereby delineating the boundary to distinguish low or negative from high HLA-DR expression in the CD33^+^CD14^+^ monocyte population.

Further complicating the comparison of CD14^+^HLA-DR^lo/neg^ monocyte levels between different studies is the output of how the cells are enumerated. Examples from the literature include reported cells as a percent of peripheral blood mononuclear cells (PBMCs), a percent of total leukocytes, a percent of monocytes, total cells per volume of blood (i.e., cells/μl), mean fluorescence intensity (MFI), molecules per cell, and based on other non-flow cytometry methods such as polymerase chain reaction assays. For flow cytometry based assays, reporting the abundance of CD14^+^HLA-DR^lo/neg^ monocytes as a percent of PBMCs or total leukocytes is the least informative, particularly when measuring these cells from cancer patients where many patients exhibit severe leukopenia and/or lymphopenia. This phenomenon leads to artificially high percentages because the comparative denominator of total PBMCs or leukocytes can be much lower than in the control or healthy volunteer subject groups. Since it remains to be determined how much HLA-DR expression must be diminished before the monocyte becomes deactivated or immunosuppressive, it may be more appropriate to measure surface expression of HLA-DR on monocytes rather than measure cell abundance. While MFI is commonly used for measuring surface expression, it is difficult to standardize MFI values between different instruments within the same laboratory let alone between different laboratories. As such, we recommend the use of fluorescent beads such as Anti–HLA-DR/Anti-Monocyte Quantibrite™ (BD Biosciences) to better assess the quantity of surface protein expression on cells. Finally, computational approaches for reducing the effect of procedural and inter-user variability on assay results have been developed which use coefficient of variation to quantify the HLA-DR spread on monocytes in healthy subjects and patients with melanoma (57).

Another source of variation that contributes to inconsistent results is the method by which blood samples are processed. The most common processing steps for the isolation of peripheral blood mononuclear cells (PBMCs) include sucrose gradient centrifugation followed by subsequent cryopreservation of the purified cells. In direct comparisons of processing steps in samples from patients with gastrointestinal cancer, Duffy et al. found that although the processing steps yielded relatively consistent results when comparing cancer patients to healthy subjects, the absolute numbers of CD14^+^HLA-DR^lo/neg^ monocytes were significantly different when comparing whole blood staining to freshly isolated PBMCs in the cancer patient cohort (128). Several groups have found that cryopreservation can negatively affect the immunosuppressive functions, enzymatic activity, and/or the abundance/distribution of MDSC subsets (61, 129, 130). Monneret et al. found that in blood samples collected in EDTA anti-coagulant tubes, HLA-DR expression was influenced both by storage time and temperature in their study of patients with sepsis and in control subjects (131). After sample collection, increased storage time at room temperature led to dramatically increased HLA-DR expression both in terms of percent positive monocytes and MFI. Higher storage temperatures also appeared to increase HLA-DR levels as well. Docke et al. also found that processing and transport steps can influence HLA-DR and thus recommended staining unprocessed blood within 4 h of the blood draw (132). Additionally, they found that the HLA-DR values for samples that were lysed/washed vs. lysed/no wash strongly correlated despite the slightly higher overall HLA-DR values reported in the lyse/no wash samples. In summary, there are many processing steps that affect the accurate measurement of CD14^+^HLA-DR^lo/neg^ monocytes. Results from minimally processed samples appear to yield the most reliable results for HLA-DR quantification. Therefore, as whole blood staining of fresh blood is becoming more standard practice, this will no doubt improve the prospect of using CD14^+^HLADR^lo/neg^ monocytes as a biomarker for understanding responses to cancer immunotherapy.

## Conclusions

There is now a large body of evidence linking CD14^+^HLADR^lo/neg^ monocytes to systemic immune suppression and paralysis and their negative affect on cancer immunotherapy. As new evidence suggests that systemic immunity plays an important role in optimal responses to cancer immunotherapy (51, 133), circulating monocytes likely contribute significantly to this phenomenon. From studies published to date, it appears that the various immunotherapeutic approaches do not drastically change the abundance of CD14^+^HLADR^lo/neg^ monocytes but their pre-treatment levels correlate with poorer or more favorable outcomes in most settings. While deciphering the precise mechanisms of CD14^+^HLADR^lo/neg^ monocyte-mediated suppression in humans will remain difficult, the established data warrant further efforts to investigate novel ways to counteract these cells. Finally, the immunosuppressive CD14^+^HLA-DR^lo/neg^ monocyte not only may be a very good therapeutic target, but also may be a very good candidate for biomarker development. They are easy to quantify, likely to reflect general systemic immunosuppression, and may even reflect what is happening in the tumor microenvironment.

## Author Contributions

AM contributed to the development, writing, and illustrations of the article. DG contributed to the study concept, development, and writing of the article. MG contributed to the study concept, development, writing, and illustrations of the article. All authors read and approved the final manuscript.

### Conflict of Interest Statement

MG has intellectual property and two patents or pending patents associated with the enumeration of immune system profiles. The remaining authors declare that the research was conducted in the absence of any commercial or financial relationships that could be construed as a potential conflict of interest.
